# Development of an electrosurgery-compatible simulation task for quantitatively assessing oral cancer resection skills: initial validity evidence

**DOI:** 10.1186/s12909-026-08743-5

**Published:** 2026-02-07

**Authors:** Kayo Sakamoto, Sohei Mitani, Naoki Nishio, Takashi Kitani, Eriko Sato, Keiko Tanaka, Toru Ugumori, Hiroyuki Wakisaka, Naohito Hato

**Affiliations:** 1 Department of Otolaryngology-Head and Neck Surgery, Ehime University Graduate School of Medicine, Shitsukawa, Toon, Ehime 791-0295 Japan; 2 Department of Otorhinolaryngology, Nagoya University Graduate School of Medicine, 65 Tsurumai-Cho, Showa Ward, Nagoya, Aichi 466-8560 Japan; 3 Department of Epidemiology and Public Health, Ehime University Graduate School of Medicine, Shitsukawa, Toon, Ehime 791-0295 Japan; 4 Ugumori ENT Clinic, 3-10-25 Yougonishi, Matsuyama, Ehime 790-0046 Japan; 5 Ehime Prefectural University of Health Sciences, 543 Takooda, Tobe, Ehime 791-2101 Japan

**Keywords:** Electrosurgery, Margin management, Oral cancer, Patient safety, Quantitative assessment, Simulation task

## Abstract

**Background:**

The quality of oral cancer resection is extremely important for patient outcomes, such as local control and survival. However, most current simulators either provide only rater-dependent feedback or are not compatible with electrosurgery. Therefore, we developed an electrosurgical simulation task for oral cancer resection that provides objective quantitative metrics and collected initial validity evidence.

**Methods:**

We developed a soft tissue simulation task using a plant-derived model that supports electrosurgery. As quantitative measures demonstrating “ensuring appropriate margins” in oral cancer resection and “maintaining safety” during electrosurgery, we employed nine-directional margin error distance and tumor bed carbonization degree measured using a spectral colorimeter. As validity evidence of the task, 10 expert surgeons completed a questionnaire about the task. In addition, five experts and 12 novices performed the task, and quantitative data obtained from their performance was used for evaluation.

**Results:**

The replication of oral cancer resection was highly evaluated (4.4 out of 5 points), and quantitative measures for evaluating the skills of surgeons (4.8 out of 5 points) were agreed upon by experts. The internal consistency of the measures was good (Cronbach's alpha: 0.803). Compared to novices, experts had smaller margin errors (0.79 mm vs 2.45 mm), lower carbonization (ΔE: 2.33 vs 8.70), faster resection times, and fewer grasping attempts (all *P* < 0.001).

**Conclusion:**

This user-friendly plant-derived simulation task is compatible with electrosurgery and provides objective quantitative performance metrics. These findings support its use as a practical assessment tool for formative feedback in simulation-based training.

**Supplementary Information:**

The online version contains supplementary material available at 10.1186/s12909-026-08743-5.

## Background

The 5-year overall survival rate of patients with oral cancer is approximately 50%, and it has not improved during the past three decades [1]. The primary treatment for oral cancer is surgery, and the quality of tumor resection is reportedly an important independent predictor of patient outcomes, such as local control and survival [2–5]. The quality of the initial tumor resection is particularly crucial, as additional resections after the tumor has been resected once lead to poor survival [6].

In oral cancer resection, a smaller margin poses the risk of local recurrence caused by microscopic tumor spread [6], whereas excessive resection or cauterization may cause swallowing or speech dysfunction and delayed wound healing [7]. In addition, monopolar electrosurgery, which has become the mainstream for oral cancer resection, while excelling in bleeding control, causes thermal damage, which alters the resection surface and makes accurate margin assessment in pathological diagnosis difficult [8]. Therefore, surgeons need to undergo training to acquire comprehensive skills, including techniques for ensuring appropriate margins and minimizing tissue damage during electrosurgical procedures, for improved quality of oral cancer resection.

Various simulation tasks using animal tissues [9] and silicone [10–12] have been developed to aid repeated practice of oral cancer resection. However, most of these tasks rely on checklists using rater-dependent ordinal variables for assessing surgical skills, and objective continuous performance measures without real-time expert raters remain limited. In addition, electrosurgery-specific assessment of thermal damage has rarely been incorporated into oral cancer resection simulation. Therefore, this study aimed to develop a new electrosurgery-compatible simulation task for oral cancer resection, which can assess surgical skills using continuous objective measures, and collect initial validity evidence for this task.

## Materials and methods

### Study design

This was a multi-phase development study designed to gather initial validity evidence of an electrosurgery-compatible simulation task for oral cancer resection. The study consisted of: (1) simulation task development, (2) measure development informed by an expert focus group [13], and (3) collection of validity evidence using expert questionnaire responses and performance data from participants completing the simulation task.

### Setting/context and ethics

The study was conducted at an academic university hospital and was approved by the Institutional Review Board of Ehime University (registration number: 2102007). Written informed consent was obtained from all participants prior to participation.

### Participants and recruitment

Participants were recruited according to the research phase as (i) experts as focus group participants to inform measure development, (ii) experts as questionnaire respondents after task implementation, and (iii) experts and novices participating in the performance study. All experts were fellowship-trained in head and neck surgery, board-certified by the Japan Society for Head and Neck Surgery, practiced at academic centers, and had each performed more than 500 head and neck surgeries. Novices were final-year medical students with no experience performing oral cancer surgery or other electrosurgical procedures.

### Development of a simulation task for oral cancer resection

**Fig. 1:**
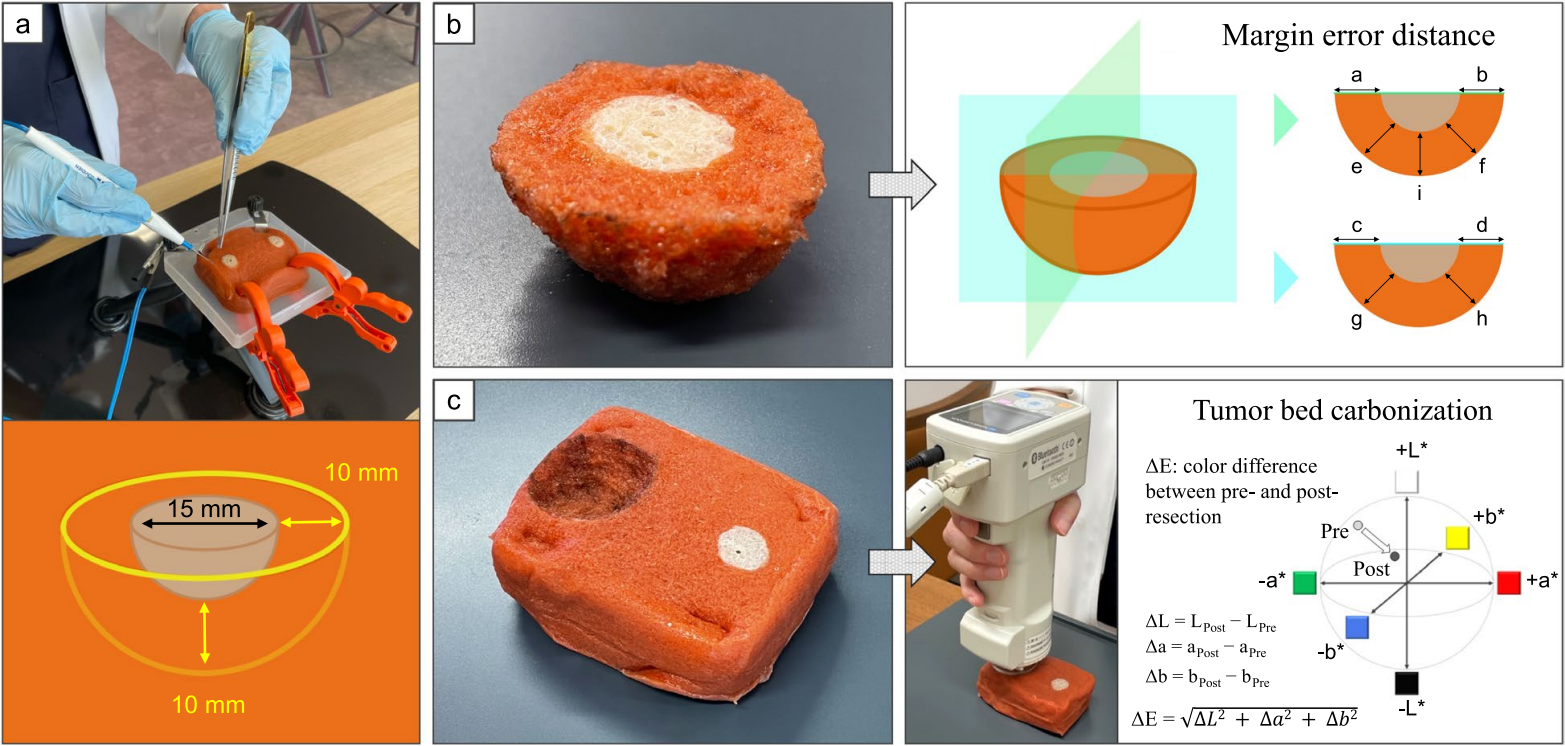
Tumor resection simulation task. **a** A simulation task was designed to ensure a 10 mm safety margin in all directions from the uniform hemispherical phantom tumor without excess or deficiency. **b** Secured tumor margins were measured in nine directions (a–i) by dividing the extracted specimen into two directions. **c** The degree of tumor bed carbonization (ΔE) during tumor resection was measured using a spectral colorimeter (CM-700d; Konica Minolta Inc., Tokyo, Japan). This difference indicated the degree of charring (carbonization) caused by electrosurgery

To develop a simulation task, we obtained surgical training models (Versatile Training Tissue [VTT]; KOTOBUKI Medical Inc., Saitama, Japan). The VTT material was konjac potato flour [14], which is derived from plants and can be easily disposed of, unlike medical waste. The material comprises glucomannan that turns black with carbonization at temperatures higher than 200 °C, similar to human tissues [15]. This characteristic enables the replication of electrosurgery, which is commonly used in oral cancer resection. The simulation task was created by modifying a commercially available VTT tumor model. The authors (K.S. and S.M.) tried prototypes using phantom tumors with uniform diameters of 10 mm, 15 mm, and 20 mm and confirmed that the 15 mm hemispherical phantom tumor was suitable for the task in terms of usability. In this model, as with epithelial tumors, which account for the majority of oral cancers, the surface of the tumor was visible, but the deeper part was buried in the tissue and could not be seen from the surface. The simulation task of tumor resection was designed to ensure a 10 mm safety margin of the phantom tumor in all directions, avoiding both excess and insufficient resection (Fig. 1a). The following standardized methods were used (Supplementary Material 1). The surgeons performed the task alone, without assistants. They manipulated a monopolar handpiece (electrosurgical pencils with stainless steel electrodes; Covidien, Dublin, Ireland) using the dominant hand and DeBakey forceps using the non-dominant hand. The VIO50C electrosurgical unit (ERBE Elektromedizin GmbH, Tübingen, Germany) was used, and the output setting selected for the unit was the coagulation mode, which operated at a power level of 15 Watts. They were allowed to use a ruler to preoperatively mark the resection margins at no more than eight points on the VTT surface, as in actual surgeries.

### Establishment of quantitative assessment method

To identify a practical assessment method, a focus group was conducted to explore the considerations experts make while performing oral cancer resections. This group included six experts from five academic institutions.. Discussion in the focus group, lasting 1 h, was moderated by another surgeon (K.S.) using a discussion guide including simple open-ended questions (Supplementary Material 2). The session was recorded, and the transcriptions were subsequently prepared. These textual materials underwent qualitative content analysis [16]. This analysis was collaboratively carried out by two board-certified head and neck surgeons (S.M. and N.N.). Initially, the surgeons meticulously reviewed the textual material to identify meaning units relevant to the research objectives. Following this, codes were assigned to all identified units, and categories were developed based on these codes. The insights on oral cancer resection derived from the focus group were organized under three categories: "preoperative planning," "precise resection of soft tissue based on planning" and "device usage in electrosurgery." From these categories, two main themes related to oral cancer resection emerged: "ensuring appropriate margins" and "maintaining safety" (Supplementary Material 3). In the focus group, the experts pointed out the difficulty in resecting according to the preoperative plan and the importance of appropriate countertraction for safe device usage in electrosurgery, indicating the need for simulation tasks that incorporate these considerations.

Based on the results of the focus group, it was determined that “ensuring appropriate margins” could be quantitatively assessed using the extracted specimens of the phantom model. By dividing the extracted specimen into two directions, the margins of the resected tumor during the simulation task were measured in nine directions (Fig. 1b). The margin assessment included four peripheral margins (a, b, c, d), four diagonal margins (e, f, g, h), and the deep margin (i); these were measured using a ruler and were recorded. The absolute value of the distance from 10 mm was defined as the margin error to evaluate whether the surgeons followed the set resection line.

On the other hand, “maintaining safety” during electrosurgery was measured by the degree of tumor bed charring (carbonization) during electrosurgery. To determine the degree of tumor bed carbonization, the spectral colorimeter CM-700d (Konica Minolta Inc., Tokyo, Japan) was used to measure the color difference (ΔE) of the tumor bed by resection (Fig. 1c). A spectral colorimeter measures the amount of light absorbed by a sample by directing a light beam through the sample, thus enabling a quantitative measurement of color difference. ΔE was defined as the distance between two points in the L*a*b* color space by the International Commission on Illumination. The lightness value, L*, represents black at 0° and white at 100°. The a* axis is relative to the green–red opponent colors, with negative values in the green area and positive values in the red area. The b* axis represents the blue–yellow opponents, with negative values in the blue area and positive values in the yellow area. ΔE was derived from the square root of the sum of the squares of the respective differences (i.e., ΔL, Δa, and Δb). The color of the non-tumor area of the VTT plane was measured as a control before tumor resection. After tumor resection, the colorimeter was placed in contact with the center of the tumor bed to measure the color of the area. The color difference between the tumor bed after resection and the control was calculated as ΔE and indicated the degree of tumor bed carbonization caused by electrosurgical tumor resection.

Additionally, the tumor resection time in the task was measured, and the number of repeated grasps using forceps was counted during the task.

### Collection of validity evidence

Validity evidence for the task was gathered using the Messick’s framework [17]. Two board-certified head and neck surgeons (S.M. and N.N.) developed a questionnaire using a Likert scale ranging from 1 (strongly disagree) to 5 (strongly agree) to evaluate the replication of oral cancer resection and the quantitative assessment methods for this study (Supplementary Material 4). The questionnaire also inquired about the convenience of the simulation task and its usefulness for oral cancer resection training. Experts completed the questionnaire after performing the simulation task.

The measurements by the experts and novices were compared to confirm any differences in the simulation task. After being informed of the tumor resection task, all participants performed the task three consecutive times in a standardized manner. The participants were observed to follow unified instructions while performing the task. The internal consistency of the four quantitative measures was calculated using Cronbach’s alpha to evaluate the internal structure.

### Statistical analysis

All data were tabulated, and statistical tests were performed using the JMP version 13 statistical software package (SAS Institute Inc., Cary, NC). Cronbach’s alpha was calculated using standardized values of the four measures. Distributional assumptions were assessed for each outcome using visual inspection (histograms and Quantile–Quantile Plots) and the Shapiro–Wilk test. Shapiro–Wilk testing suggested that margin error distance did not significantly deviate from normality, whereas tumor bed carbonization, resection time, and number of repeated grasps showed departures from normality (*P* < 0.05). Given the small sample sizes and the non-normal distribution of several outcomes, we used non-parametric tests for between-group comparisons for consistency across outcomes. Specifically, the Mann–Whitney U test was used to compare the scores of experts with those of novices. The coefficient of variation (CV) was defined as the standard deviation (SD) divided by the mean value. The F-test was performed to assess the difference between the CV of the experts and that of the novices for each task. Statistical significance was set at *P* < 0.05; results are interpreted as exploratory due to the small sample size. Values are reported as mean ± SD unless stated otherwise.

## Results

### Evaluation of the simulation task based on expert questionnaires

After performing the simulation task, 10 experts from eight institutions completed the questionnaire evaluating the replication and quantitative measures (Table 1). All of them were male, and their median age was 42 years (range, 36 − 54 years). The replication of oral cancer resection was rated highly (4.40). Furthermore, the surgeons agreed with the four quantitative measures used to assess surgeons' skills (4.80). In terms of questionnaire feedback, the surgeons highly valued the convenience of the simulation task (4.90), and all of them rated the usefulness of the task for training for oral cancer resection with the highest score of 5 points.

**Table 1:** Expert responses to a 5-point Likert scale

Questions		Mean	SD
Replication	Overall	4.40	0.52
	Resection of soft tissue	4.60	0.52
	Device usage in electrosurgery	4.70	0.48
Quantitative measures	Overall	4.80	0.42
	Margin error distance of extracted specimens (ensuring appropriate margins)	4.80	0.42
	Tumor bed carbonization (maintaining safety)	4.60	0.52
	Tumor resection time	4.70	0.48
	Number of repeated grasps using forceps	4.60	0.52
Others	Convenience of the simulation task	4.90	0.32
	Usefulness as training for oral cancer resection	5.00	0.00

*SD* Standard deviation

### Evaluation based on participants’ performance in the simulation task

**Fig. 2:**
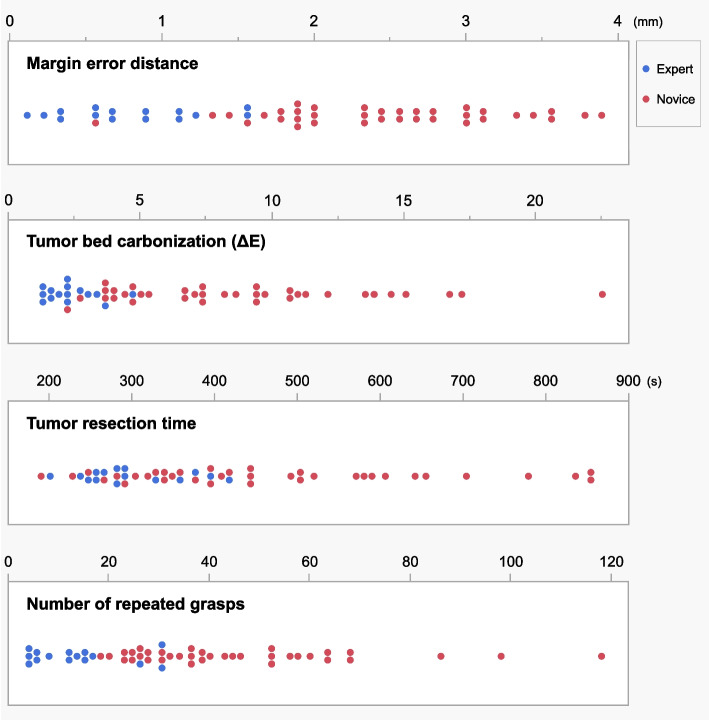
Performance dashboard for experts and novices. Individual data are shown for margin error distance, tumor bed carbonization (ΔE), tumor resection time, and number of repeated grasps. Lower values typically indicate better performance for all metrics

Five experts from four institutions and 12 novices participated in the performance study. All participants were confirmed to follow the instructions while performing the task. The mean values for the four measures across all participants were as follows: margin error distance, 1.96 ± 1.03 mm; tumor bed carbonization (ΔE), 6.83 ± 4.94; tumor resection time, 416 ± 175 s; and number of repeated grasps, 37 ± 24. The internal consistency of the four measures was good (Cronbach’s alpha: 0.803). Overall performance differed between groups, as experts exhibited smaller margin errors, lesser carbonization, shorter resection times, and fewer repeated grasps compared to novices (Table 2). The dashboard in Fig. 2 displays individual data to illustrate the distribution of performance.

**Table 2:** Participants and performance in the simulation task

		Expert	Novice	
		*n* = 5	*n* = 12	*p*-value
Age (years, range)		49 (39–53)	24 (23–26)	
Sex (male/female)		5/0	4/8	
Measures (mean ± SD)	Margin error distance (mm)	0.79 ± 0.46	2.45 ± 0.76	< 0.0001
	Tumor bed carbonization	2.33 ± 0.96	8.70 ± 4.71	< 0.0001
	Tumor resection time (s)	295 ± 63	466 ± 183	0.0005
	Number of repeated grasps	13 ± 9	45 ± 23	< 0.0001

*SD* Standard deviation

**Fig. 3:**
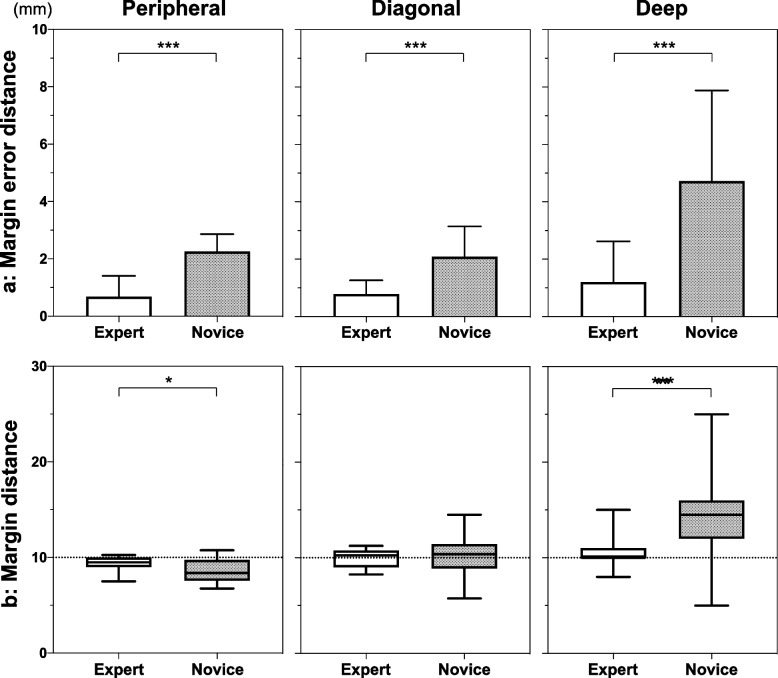
Margin distance and margin error distance. **a** Margin error distance (absolute distance from 10 mm). The bars represent the mean and standard deviation. **b** Margin distance from tumor to edge. The box extends from the 25th to 75th percentiles. The line in the middle of the box represents the median. The whiskers represent the minimum to maximum. *P < 0.05, **P < 0.01, ***P < 0.001

The absolute distance of the margin errors in all directions of the experts was significantly shorter than that of the novices (Fig. 3a). There was a significant difference in the deep margins achieved by the experts and novices (1.20 ± 1.42 mm vs. 4.72 ± 3.16 mm; *P* < 0.0001). The overall mean margin distances achieved by the experts and novices were not significantly different (*P* = 0.57). However, the CV of the experts was significantly lower than that of the novices (0.08 vs. 0.16; *P* = 0.0023). The experts secured stable 10 mm margins in all directions, whereas the novices performed resection with longer deep margins (13.83 ± 4.22 mm, *P* = 0.0014) (Fig. 3b).

**Fig. 4:**
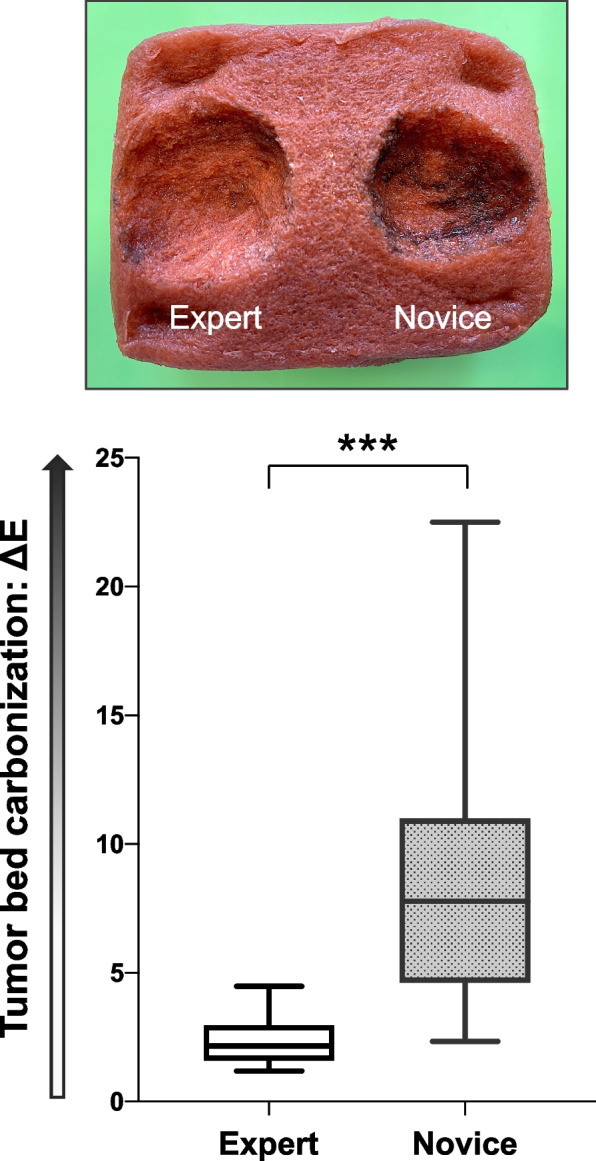
Tumor bed carbonization caused by electrosurgical tumor resection. The image shows the tumor bed carbonization after resection (ΔE) by an expert and a novice. The box-and-whisker plot is the same as that shown in Fig. 3b. ***P < 0.001

The spectral colorimeter detected a smaller ΔE after completion of the task by the experts, indicating a lesser degree of tumor bed carbonization achieved by the experts than by the novices (2.33 ± 0.96 vs 8.70 ± 4.71; *P* < 0.0001) (Fig. 4).

## Discussion

We developed a new electrosurgery-compatible simulation task that can quantitatively assess surgeons’ oral cancer resection skills and provided initial evidence of its validity. In this study, a focus group facilitated the articulation of experts’ understanding of oral cancer resection into two primary themes: “ensuring appropriate margins” and “maintaining safety.” Based on these insights, the quantitative assessment method was established. The validity evidence of the task was gathered from questionnaire responses and quantitative measurements obtained by performing the task.

We developed a simulation task for oral cancer resection, and the task demonstrated high replication of soft tissue resection and device usage in electrosurgery. While silicone [10–12] and plastic [18] are commonly used to simulate soft tissues, they are incompatible with electrosurgical instruments. In contrast, the VTT phantom material used in this study not only reproduces soft tissues but also supports electrosurgical instruments that are prevalent in oral cancer resection. Furthermore, our simulation task using this VTT model was highly rated for convenience. Since VTT is derived from plants, the model is much easier to handle in terms of ethics, management, and disposal compared to cadavers [19] or porcine tongues [9], which are conventionally used as simulation models for oral cancer.

Ensuring the quality of surgical training is crucial for providing patients with safe and high-quality care. However, a scoping review [20] of 68 surgical training studies indicates that surgical training lacks quantitative assessment beyond the number of surgical operations, underscoring the need for clear and measurable outcome indicators. In simulation training, a systematic review in plastic surgery [21] revealed that, among 116 studies, only 27% and 24% provided sufficient evidence for content and internal structure validity in the Messick’s framework, respectively, highlighting the importance of validity research. In this study, the four quantitative measures, including “ensuring appropriate margins” and “maintaining safety,” established through a focus group were considered suitable for quantifying oral cancer resection skills, and the internal consistency was good. Existing surgical skill assessment scales, such as Global Rating Scale [22] and Global Operative Assessment of Laparoscopic Skills [23], rely on judgment of evaluators, whereas recent advancements in computer navigation [24] and motion capture [25, 26] offer the potential for numerical feedback. Nevertheless, these methods also face challenges such as the high cost of equipment, making widespread implementation difficult. By contrast, the quantitative assessment items used in this study offer numerical feedback while maintaining high usability. Referencing dashboards of distinct metrics (Fig. 2) allows learners and instructors to quickly identify metric-specific strengths and weaknesses, there by facilitating formative feedback.

When attempting to maintain a 10 mm margin, resection of the deep portion is technically more difficult than that of the peripheral portion, as indicated by the quantitative results. In addition to frozen section assessments during surgery, fluorescence imaging [27] and three-dimensional specimen scanning [28] have been reported to assess intraoperative secured margins in recent years. However, because these intraoperative assessments were based on the specimen after resection, additional resection cannot be avoided when the intraoperatively assessed margin is positive. An initially positive margin eventually revised to negative has been associated with adverse outcomes, such as local recurrence and poor survival [6]. Training focused on the deep margin could help surgeons learn the technique of initial resection to ensure adequate margins and avoid additional resection and could improve local control and survival for patients with oral cancer.

Carbonization after tumor resection was more frequent in novices. Although one aim of electrosurgery most surgeons use for oral tumor resection is coagulation for adequate hemostasis, blackened tissue coagulated by carbonization is not a hallmark of meticulous dissection. This coagulum causes rebleeding to occur more easily with the detachment of incrustation [29]. From an oncological point of view, unnecessary thermal damage should also be avoided because thermal injury artifacts negatively affect the margin status, increasing the probability of recurrence [8]. Carbonization is often related to inadequate countertraction by the non-dominant hand [30], and expert surgeons regard countertraction as an essential skill in head and neck surgery [31]. Therefore, emphasizing countertraction during repeated practice may help reduce carbonization and support safer electrosurgical handling. In addition, novices tended to have longer resection times and a greater number of repeated grasps; improving countertraction with the non-dominant hand may also enhance efficiency and reduce corrective maneuvers.

This study had some limitations. First, the shape of the phantom tumor was defined as hemispherical, and the color was distinct; however, the actual tumors often have irregular or asymmetrical shapes and are sometimes not clearly visible and extend deeper than they appear, requiring palpation during surgery. In addition, the narrow surgical field of the oral cavity may limit the movement of surgical devices. However, even if the tumor is hemispherical in an open space, confirmation of the margin in the deep portion is not easy as the results indicated. Therefore, incorporating this simple task into training may help learners practice executing an intended resection margin and improve technical precision. However, determining where the oncological appropriate resection should be performed remains a clinical judgment that must be developed in vivo under senior supervision. Second, this study evaluated the skills of experts and novices and did not perform a stepwise evaluation of surgeons' experience. Further research should include the stepwise growth of surgeons practicing this simulation task. Practicing this task, including the quantitative visualization of skills, could lead to more effective learning by providing feedback on the surgeons' ongoing strengths and weaknesses. Third, due to the small sample size of participants, the results are exploratory, and there is also concern about the potential for social desirability bias. Future work should expand validity evidence by examining response process (e.g., video review of task performance and post-task interviews). It should also evaluate consequences of use through standard setting, decision thresholds, and transfer to clinical performance, and confirm reproducibility and generalizability in larger, multi-institution samples.

## Conclusions

We developed an electrosurgery-compatible simulation task for oral cancer resection and gathered initial validity evidence to support the intended interpretation. This task provides objective performance metrics that can be used to assess technical execution and support formative feedback in simulation-based training.

## Supplementary Information

Supplementary Material 1. An oral cancer simulation task. A simulation task for oral cancer resection designed to simulate resection of soft tissue using an electrosurgical device.

Supplementary Material 2. A discussion guide for the focus group. The focus group was conducted using the discussion guide.

Supplementary Material 3. Topics on oral cancer resection raised during an expert focus group. The qualitative content analysis of the focus group helped organize the considerations made by experts while performing oral cancer resections.

Supplementary Material 4. A questionnaire developed for this study to evaluate the simulation task. The questionnaire developed specifically for this study to evaluate the replication of oral cancer resection, the quantitative assessment methods, usability, and educational utility using a 5- point Likert scale.

## Data Availability

Data that support the findings of this study are available on request from the corresponding author, SM. Data are not publicly available owing to their containing information that can compromise the privacy of research participants.

## Abbreviations

CV: Coefficient of variation
SD: Standard deviation
VTT: Versatile Training Tissue
