# Supplementary material for: Development of an electrosurgery-compatible simulation task for quantitatively assessing oral cancer resection skills: initial validity evidence
Source: BMC Med Educ. 2026 Feb 7;26:408. doi: 10.1186/s12909-026-08743-5 (PMC12977834; doi:10.1186/s12909-026-08743-5)
Supplement: Supplementary file 2 — Supplementary Material 2. A discussion guide for the focus group. The focus group was conducted using the discussion guide. [file 12909_2026_8743_MOESM2_ESM.pdf]

Supplementary Material 2.

Discussion Guide for Focus Group

The purpose of this focus group is to elucidate the expert insights and experiential knowledge to developing simulation tasks for oral cancer resection.

1. What, in your view, constitutes a “good” oral cancer surgery?

2. What do you consider to be the key factors or strategies for performing a high-quality oral cancer surgery in actual clinical practice?

3. How did you acquire or learn the surgical techniques you currently use?

4. Please tell us about the devices you use. What aspects do you consider important or challenging when using them?

That concludes our questions. However, if there is anything else you feel we have not covered or any additional points you would like to discuss, please feel free to share.
