# Supplementary material for: Development of an electrosurgery-compatible simulation task for quantitatively assessing oral cancer resection skills: initial validity evidence
Source: BMC Med Educ. 2026 Feb 7;26:408. doi: 10.1186/s12909-026-08743-5 (PMC12977834; doi:10.1186/s12909-026-08743-5)
Supplement: Supplementary file 3 — Supplementary Material 3. Topics on oral cancer resection raised during an expert focus group. The qualitative content analysis of the focus group helped organize the considerations made by experts while performing oral cancer resections. [file 12909_2026_8743_MOESM3_ESM.pdf]

Supplementary Material 3

Theme	Categories	Fre	Subcategories	Fre	Codes	Fre	Examples
Ensuring adequate margins	Preoperative planning	29	Resection without excess or deficiency	14	Striving for resection without excess or deficiency	4	Achieving resection without excessive removal or overly close margins as planned preoperatively
					Functional impairment caused by excessive resection	3	Excessive resection may lead to functional impairment
					Reduced curability due to insufficient margins	7	Margins that are too close may result in residual tumor
			Setting the resection line preoperatively	15	Assessment of the extent of tumor invasion	5	Accurate evaluation of the extent of tumor invasion and planning
					Determining composite tissue for combined resection	7	Resection of bone when bone invasion is present
					Simulation of surgical steps	3	Simulating all tools and scenarios to be used
	Precise resection of soft tissue based on planning	58	Resection as envisioned preoperatively	52	Challenges in estimating the distance from the tumor	21	Following the preoperative plan is the most challenging aspect
					Difficulty in securing deep margins	11	Cutting deep areas with a three-dimensional image in mind is difficult
					Angle of the device	9	Shallow angles of the electrocautery can lead to closer deep margins
					Changes in resection lines due to soft tissue countertraction	11	Soft tissue shapes and positions change with countertraction, increasing difficulty
			Modification of the resection line based on intraoperative judgment	6	Responding to deviations from simulation	6	Although procedures generally follow the simulation, changes are necessary in cases of unexpected tumor progression
Maintaining safety	Device usage in electrosurgery	29	Appropriate countertraction	29	Resection without collateral damage	5	Proper tension allows for early detection and appropriate handling of blood vessels
					Minimizing tissue charring during resection	8	Tissue charring can damage tissue and make margin evaluation difficult
					Efficient dissection	11	Without proper tension, effective cutting becomes difficult
					Addressing changes in tissues caused by dissection	5	The direction of countertraction changes between the start and end of cutting, even for the same tissue

Fre: Frequency represents the number of times the topic has been brought up in the discussion.
