# Supplementary material for: Development of an electrosurgery-compatible simulation task for quantitatively assessing oral cancer resection skills: initial validity evidence
Source: BMC Med Educ. 2026 Feb 7;26:408. doi: 10.1186/s12909-026-08743-5 (PMC12977834; doi:10.1186/s12909-026-08743-5)
Supplement: Supplementary file 4 — Supplementary Material 4. A questionnaire developed for this study to evaluate the simulation task. The questionnaire developed specifically for this study to evaluate the replication of oral cancer resection, the quantitative assessment methods, usability, and educational utility using a 5- point Likert scale. [file 12909_2026_8743_MOESM4_ESM.pdf]

**Supplementary Material 4**

## Expert Evaluation Questionnaire
## Electrosurgery-Compatible Simulation Task for Oral Cancer Resection

**Date (YYYY-MM-DD): __________________**

**Instructions:** We have developed a surgical simulation task for oral cancer resection, intended for novice surgeons. Please complete this questionnaire immediately after performing the simulation task. Rate each statement on a 5-point Likert scale where 1 = Strongly disagree and 5 = Strongly agree. If an item does not apply or you are unsure, select 3 (Neither agree nor disagree).

### Participant Information

Institution: __________________
Board certification in Head & Neck Surgery (year): _________
Years since certification: _________
Estimated number of head & neck surgeries performed: _________
Age (years): _________
Sex: ◻ Male ◻ Female ◻ Other ◻ Prefer not to say

**Response scale:** 1 = Strongly disagree, 2 = Disagree, 3 = Neither agree nor disagree, 4 = Agree, 5 = Strongly agree

### Section A. Replication of Oral Cancer Resection

| Statement | 1 | 2 | 3 | 4 | 5 |
|---|---|---|---|---|---|
| The simulation realistically replicates soft tissue resection in oral cancer surgery. | ◻ | ◻ | ◻ | ◻ | ◻ |
| The simulation allows realistic use of monopolar electrosurgical instruments. | ◻ | ◻ | ◻ | ◻ | ◻ |
| Overall, the | ◻ | ◻ | ◻ | ◻ | ◻ |

| | | | | | |
|---|---|---|---|---|---|
| simulation appropriately replicates oral cancer resection. | | | | | |

**Section B. Suitability of Quantitative Measures**

| Statement | 1 | 2 | 3 | 4 | 5 |
|---|---|---|---|---|---|
| The 'margin error distance (nine-directional)' is an appropriate metric for ensuring appropriate margins. | □ | □ | □ | □ | □ |
| The 'tumor bed carbonization (ΔE)' is an appropriate metric for maintaining safety in electrosurgery. | □ | □ | □ | □ | □ |
| Resection time is an appropriate metric for assessing performance. | □ | □ | □ | □ | □ |
| Number of repeated tissue grasps with forceps is an appropriate metric for assessing performance. | □ | □ | □ | □ | □ |
| Overall, the | □ | □ | □ | □ | □ |

selected quantitative measures are appropriate to evaluate surgeons' skills.

**Section C. Usability and Educational Utility**

| Statement | 1 | 2 | 3 | 4 | 5 |
|---|---|---|---|---|---|
| The simulation task is convenient to set up and perform. | ☐ | ☐ | ☐ | ☐ | ☐ |
| The simulation is useful for training in oral cancer resection. | ☐ | ☐ | ☐ | ☐ | ☐ |

**Open-ended comments (optional)**

____________________________________________________________

Thank you for your participation.
